# New N-Terminal Fatty-Acid-Modified Melittin Analogs with Potent Biological Activity

**DOI:** 10.3390/ijms25020867

**Published:** 2024-01-10

**Authors:** Sheng Huang, Guoqi Su, Shan Jiang, Li Chen, Jinxiu Huang, Feiyun Yang

**Affiliations:** 1 Animal Nutrition Institute, Chongqing Academy of Animal Science, Chongqing 402460, China; stevenhouse@alu.cqu.edu.cn (S.H.); chenliyouxiang8@163.com (L.C.); 2 Key Laboratory of Pig Industry Sciences, Ministry of Agriculture, Chongqing 402460, China

**Keywords:** melittin, N-terminal fatty acid conjugation, antimicrobial activity, hemolysis, proteolytic stability

## Abstract

Melittin, a natural antimicrobial peptide, has broad-spectrum antimicrobial activity. This has resulted in it gaining increasing attention as a potential antibiotic alternative; however, its practical use has been limited by its weak antimicrobial activity, high hemolytic activity, and low proteolytic stability. In this study, N-terminal fatty acid conjugation was used to develop new melittin-derived lipopeptides (MDLs) to improve the characteristics of melittin. Our results showed that compared with native melittin, the antimicrobial activity of MDLs was increased by 2 to 16 times, and the stability of these MDLs against trypsin and pepsin degradation was increased by 50 to 80%. However, the hemolytic activity of the MDLs decreased when the length of the carbon chain of fatty acids exceeded 10. Among the MDLs, the newly designed analog Mel-C8 showed optimal antimicrobial activity and protease stability. The antimicrobial mechanism studied revealed that the MDLs showed a rapid bactericidal effect by interacting with lipopolysaccharide (LPS) or lipoteichoic acid (LTA) and penetrating the bacterial cell membrane. In conclusion, we designed and synthesized a new class of MDLs with potent antimicrobial activity, high proteolytic stability, and low hemolytic activity through N-terminal fatty acid conjugation.

## 1. Introduction

In recent years, high infection and mortality rates associated with pathogenic bacteria, especially drug-resistant strains, have severely threatened global human health and livestock breeding [1,2]. Various pathogenic bacteria necessitate the wide use of antibiotics; however, the abuse of antibiotics has triggered the emergence of drug resistance in pathogenic bacteria [3]. The exploitation of novel antibiotic substances is urgently needed to resolve this problem. Antimicrobial peptides (AMPs), also known as host defense peptides, are an essential part of the innate immune system; they are used by organisms to defend against attacks from pathogens [4]. AMPs are widely distributed in microorganisms, animals, and other organisms, and they have antibacterial [5], antiviral [6], anticancer [7], and immunomodulatory [8] activities. AMPs represent the most promising antibiotic substitutes based on their rapid and broad-spectrum antibacterial effects and non-specific membrane destruction mechanisms [9].

Melittin (Mel), a small cationic linear peptide composed of twenty-six amino acid residues (GIGAVLKVLTTGLPALISWIKRKRQQ-CONH2), is a significant element in the venom of honeybees [10]. It has a variety of biological actions including antibacterial [11], anticancer [12], and antiviral [13] activities. In the molecular structure of melittin [14], two amino residues at the N-terminal and four amino residues at the C-terminal have positive charges, so the whole melittin structure has six positive charges. In addition, six amino residues at the C-terminal are hydrophilic. In addition, most of the twenty amino residues at the N-terminal are hydrophobic amino acids, and the whole peptide is amphiphilic [15]. The hydrophobic part of melittin can bind to the phospholipid bilayer membrane of a cell or bacteria, and the hydrophilic part can be free in the solution environment [16]. This amphiphilic structure is the basis for the bactericidal realization of melittin [17]. The results of a previous study showed that melittin exhibits a potent antibacterial effect against *S. aureus* at concentrations significantly below toxic concentrations; it is effective against planktonic and biofilm-embedded MRSA strains at concentrations ranging from 0.12 to 4 μM [18]. Therefore, melittin is an up-and-coming candidate for developing new antibiotics to treat pathogenic bacterial infections.

Despite its multiple potential biological activities, the applications for melittin are limited due to its severe hemolytic activity and low proteolytic stability. Melittin results in 50% hemolysis of human red blood cells at a concentration of 2 μM and 100% at 7 μM [19]. It contains two arginine and three lysine residues sensitive to trypsin; trypsin degrades melittin rapidly, and almost no melittin is left after 60 min of incubation [20].

Multiple strategies have been applied to develop novel AMPs with potent antimicrobial activity, lower cytotoxicity, and higher proteolytic stability. The replacement of unnatural amino acids with, for example, D-form and N-methyl amino acids, cyclization, polyethylene glycol (PEG) modification, and fatty acid conjugation, has been used for precursor modification and construction of antibacterial peptide drugs [21,22,23,24]. Among these methods, fatty acid conjugation could significantly tune the antibacterial activity and proteolytic stability of AMPs [25]. Zhong et al. showed that conjugation with a fatty acid on the side chain of the D-amino acid of anoplin enhanced its antimicrobial potency against multidrug-resistant bacteria [26]. Liu et al. reported that fatty acid conjugation with anoplin developed a series of lipopeptide analogs. The stability of these lipopeptides against trypsin and chymotrypsin degradation was increased 10^4^–10^6^ times [22]. It was demonstrated that fatty acid conjugation can significantly improve various bioactivity properties of peptides, including antimicrobial potency and proteolytic stability.

This study used N-terminal fatty acid conjugation to develop novel MDLs with enhanced antimicrobial activity, reduced hemolytic activity, and resistance toward proteolytic degradation. Furthermore, the antimicrobial mechanism of action of these new MDLs was investigated using membrane permeabilization and LPS/LTA competitive inhibition assays. Overall, our results indicate that fatty acid conjugation is a promising method to improve natural melittin’s antimicrobial activity and proteolytic stability.

## 2. Results and Discussion

### 2.1. Characterization of MDLs

**Figure 1:**
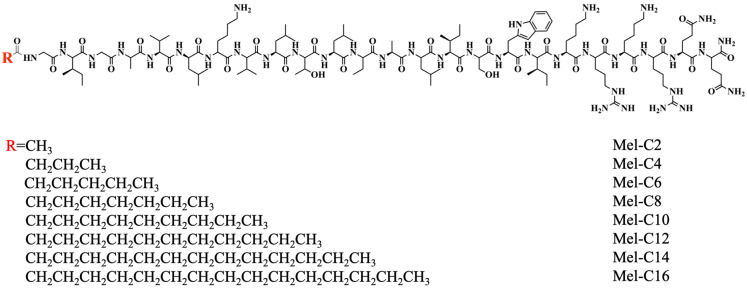
The structural formulas of MDLs. **R** (red) means alkyl chain of fatty acids with different chain lengths.

In this study, we aimed to design and synthesize a new series analog of melittin with potent antimicrobial activity, low hemolytic activity, and high proteolytic stability. Fatty acids of various chain lengths ranging from C2 to C16 were linked to the N-terminal of the melittin sequence. The structural formulas of MDLs are shown in Figure 1.

All MDLs were synthesized using solid-phase synthesis and characterized using electrospray ionization mass spectrometry (ESI-MS) and reversed-phase high-performance liquid chromatography (RP-HPLC). The sequences, molecular weights (MWs), and retention times (RTs) of these MDLs are listed in Table 1.

**Figure 2:**
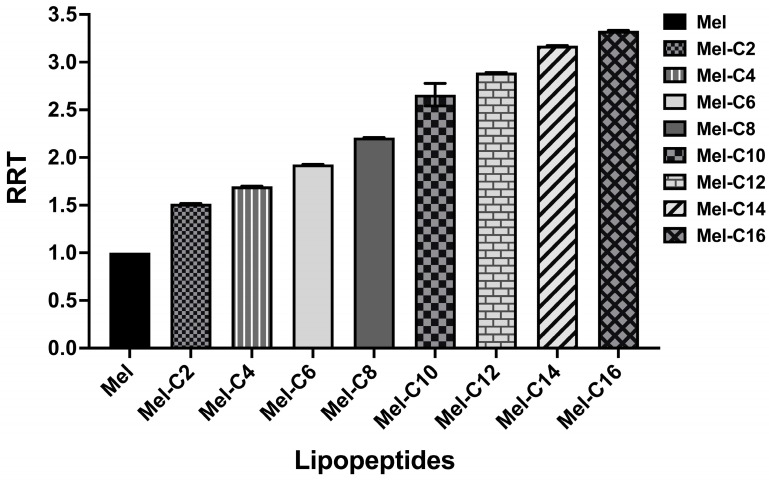
The relative retention time (RRT) of each MDL. The RT of each MDL was recorded using RP-HPLC analysis and was used to calculate the relative retention time (RRT) with melittin. The data were collected from three independent experiments, and the vertical bars represent the standard error of the mean (SEM).

The hydrophobicity, amphipathicity, and positive charge distribution of lipopeptides are crucial for their antimicrobial activities [27]. Among these, hydrophobicity has been deemed essential for promoting a hydrophobic interaction with the lipid bilayer, leading to membrane disruption, cytoplasm leakage, and eventual cell death [28]. Lipopeptides with different peptide sequences and chain lengths usually show different hydrophobicities, generating different RTs in the RP-HPLC analysis. The more hydrophobic the lipopeptide, the longer the RT on the column [29]. The RT of each MDL was recorded and is shown in Table 1. These data were used to calculate the relative retention time (RRT) with melittin, as exhibited in Figure 2.

These results indicate that with longer fatty acids covalently bound to melittin, the RRTs of the MDLs gradually increase. A similar finding was reported in a previous study; Liu et al. designed a series of N-methylated lipopeptides of anoplin by conjugating fatty acids with various chain lengths (C8~C14). Their results showed that an increase in RRT was associated with the length of the conjugated fatty acids [22].

### 2.2. Secondary Structure of MDLs

**Figure 3:**
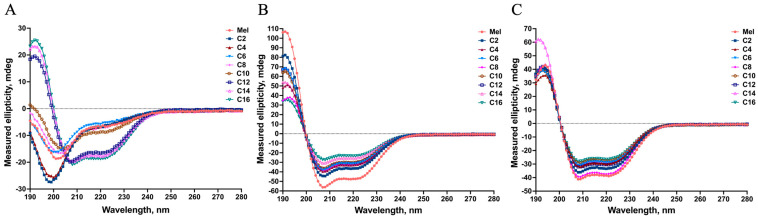
The CD spectra of the MDLs in different solution environments. The mean residue ellipticity was plotted against the wavelength. The different solution environments included (**A**) MDLs in PBS solution, which was used to mimic the physiological environment; (**B**) MDLs in 50% TFE solution, which was used to mimic the hydrophobic environment of the bacterial membrane; and (**C**) MDLs in 25 mM SDS solution, which mimicked the anionic bacterial membrane environment.

The secondary structure of antimicrobial peptides plays a vital role in their biological activity in relation to disturbing cell membranes [30]. Here, the secondary structures of MDLs were determined by circular dichroism (CD) spectroscopy under different environments, as shown in Figure 3.

The CD spectra shown in Figure 3A indicate that melittin and the MDLs with a C2~C10 tail adopted a definite random-coil conformation in phosphate-buffered saline (PBS), and the MDLs with a C12~C16 tail formed an α-helical conformation. This result agrees with an early report indicating that fatty acid conjugation can increase the α-helix content of the lipopeptide structure [31]. In 50% 2,2,2-trifluoroethanol (TFE) or 25 mM sodium dodecyl sulfate (SDS) (Figure 3B,C), the MDLs displayed a typical α-helical conformation. This is shown by one positive dichroic band at 190 nm and double-negative dichroic bands at 208 and 222 nm. To further elaborate on the CD data, the α-helix and β-fold contents were calculated and are displayed in Table 2.

The MDLs exhibited different α-helical contents in 50% TFE. As shown in Table 2, the α-helical contents of the MDLs were less than that of the parent peptide melittin. Additionally, the α-helical contents of the MDLs tended to decrease with the growth of fatty acid carbon chains. Moreover, in the presence of 25 mM SDS, the α-helical contents tended to decrease with the increased length of the fatty acid chain. In contrast, the β-fold contents of the MDLs showed an increasing trend. Similar findings were reported in a previous study [32]. Previous work has shown that AMPs display an α-helical formation in the membrane environment, have a greater affinity for anionic membranes, and demonstrate enhanced incorporation into lipid membranes, further increasing their antimicrobial activity [33]. In our study, the CD spectrum results showed that the MDLs could form more typical α-helical structures in the membrane mimetic environments (in SDS and TFE solutions) compared with the PBS solution. This indicates that the propensity of the MDLs to form an amphipathic α-helical structure might play an essential role in killing bacteria. In addition, the hydrophobicity of the MDLs conjugated with fatty acids positively correlated with the α-helical structure [34]. However, as is shown in Table 2, with the increase in the length of the fatty acids of MDLs in the membrane mimetic environments, the α-helical structure was reduced whereas the β-fold structure showed the opposite trend. Thus, the enhanced antimicrobial activity of the MDLs was most likely due to the increase in hydrophobicity due to the conjugated fatty acids, while the α-helical formation played a secondary role.

### 2.3. Antimicrobial Activity In Vitro

Recently, multiple studies have found that coupling a fatty acid to antimicrobial peptides could modify both the activity and selectivity of the peptide and the length of the fatty acid was found to be directly correlated to the antimicrobial activity of the fatty-acid-conjugated antibacterial peptide [35,36,37]. Chu-Kung et al. detected the antimicrobial activity, structure, and solution assembly properties of peptide AKK (YGAAKKAAKAAKKAAKAA) conjugated with fatty acids of different carbon chain lengths. Their results showed that the antimicrobial activity of the conjugated peptide increased with the carbon chain lengths of fatty acids up to 16 carbon atoms [38]. Here, we designed and synthesized a set of MDLs by coupling different-length fatty acids to the N-terminus of melittin to reinforce its antimicrobial activity. The antimicrobial activity against both standard and multidrug-resistant strains was assessed and is presented in Table 3. The results demonstrate that the MDLs exerted broad antimicrobial potency, and fatty acid conjugation led to length-dependent antimicrobial activity. As is shown in Table 3, when the length of the conjugated fatty acid chain ranged from 2 to 10 carbon atoms, there was a remarkable fatty-acid-chain-length-dependent increase in antimicrobial activity, with decreased minimal inhibitory concentration (MIC) values. However, the opposite effects were observed when the conjugated fatty acid chain length was more than 10 carbon atoms. Here, the hydrophobicity of the MDLs played a decisive role in their antimicrobial activity, which was significantly affected by the length of the conjugated fatty acid chain [39]. The retention time of peptides on RP-HPLC is related to their hydrophobicity, and an increase in the retention time implies an enhanced hydrophobicity [40]. According to Table 1 and Table 3, within a certain range (C2~C8), the hydrophobicity of the MDLs was highly positively correlated with their antimicrobial activity. This likely explains why the antimicrobial activity increased in a fatty-acid-chain-length-dependent manner. These results indicate that hydrophobicity is a crucial factor affecting the antimicrobial activity of MDLs. This is consistent with the proposed antimicrobial mechanism: the MDLs gather on the surface of the bacterial membrane and reach a threshold value; then, the hydrophobic residues insert into the hydrophobic region of the lipid bilayer and form pore/ion channels in the cytoplasmic membrane; this ultimately leads to bacterial death [41]. In this study, it is noteworthy that the relationship between hydrophobicity and antimicrobial activity showed a similar threshold, such that hydrophobicity above the threshold caused peptides to become too hydrophobic, thus limiting the further enhancement of antimicrobial activity [42]. Moreover, our results agree with a previous report that reduced antimicrobial activity at longer carbon chain lengths is primarily due to the self-assembly of fatty-acid-conjugated melittin [43].

### 2.4. Hemolytic Assay

Melittin is an amphipathic peptide that can interact with the red blood cell membrane, disrupt phospholipid bilayers, and exert significant hemolytic activity [44]. Therefore, reducing the hemolysis of melittin is critical in terms of its clinical application.

**Figure 4:**
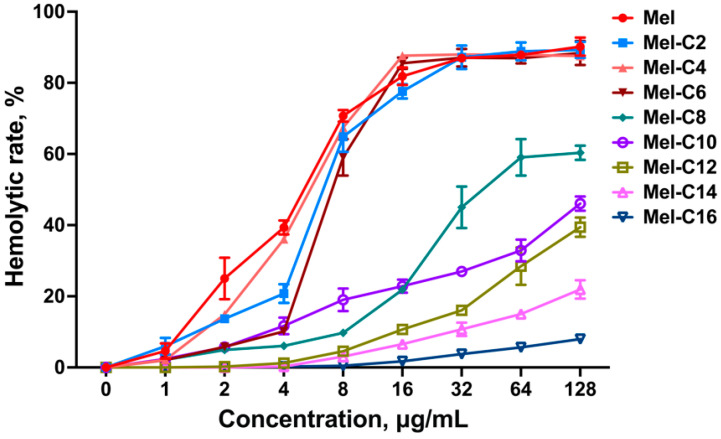
The hemolytic activity of MDLs against fresh swine erythrocytes. Hemolytic activity is based on 4% hemolysis of swine erythrocyte in PBS after 1 h incubation at 37 °C. Data represent the average ± SEM of three independent experiments.

In this study, a hemolytic assay was used to assess the MDLs by measuring the percentage of hemoglobin released from lysed swine erythrocytes after treatment for 1 h, and the results are shown in Figure 4. The hemolytic activity of the MDLs was associated with the chain length of the coupled fatty acid. Like melittin, when the concentration was more than 4 μg/mL, the hemolysis rate of the MDLs with a C2~C8 tail rapidly increased. This agrees with a previous study showing that fatty acid conjunction can increase the hydrophobicity of AMPs and intensify their membrane interaction and final disruption [45]. Grimsey et al. found that the hemolytic activity of the lipidation of short cationic antimicrobial peptides correlated with the length of the conjugated fatty acid and represented a clear correlation with hydrophobicity [24]. Previous research has shown that highly hydrophobic peptides exhibit considerable cytotoxicity toward eukaryotic cells resulting in a non-specific interaction with any cell membrane [38]. Interestingly, in this study, when the length of the fatty acid carbon chain exceeded ten, the hemolysis rate of the MDLs decreased with the increase in the length of the fatty acid chain. When the number of carbon atoms in the fatty acid chain reached 16, the lipopeptide did not show hemolysis, even at concentrations of up to 128 μg/mL. The diminished hemolysis rate may be attributed to the tendency for longer fatty acid chain lengths to promote lipopeptide self-assembly, which reduces the interaction between the lipopeptide and the erythrocyte membrane.

### 2.5. Stability Assay

#### 2.5.1. Protease Resistance Assay

**Figure 5:**
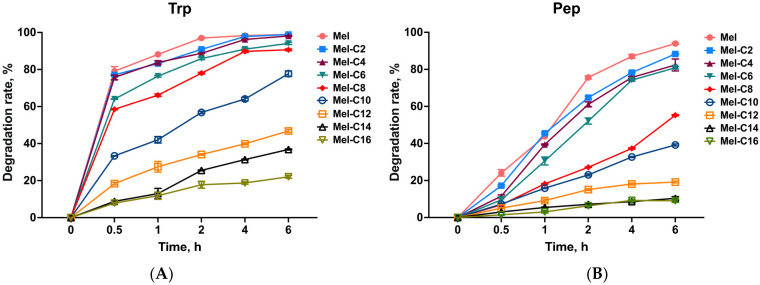
Stability of MDLs in trypsin (**A**) and pepsin (**B**) solutions. Each MDL was mixed with equal volumes of trypsin or pepsin solution and incubated at 37 °C for 6 h. Aliquots of 50 μL were withdrawn at 0, 0.5, 1, 2, 4, and 6 h, and RP-HPLC was used to detect the enzymatic digestion of the MDLs. Data represent the average ± SEM of three independent experiments.

Poor protease stability is one of the main factors that has limited melittin’s clinical application. Previous research has demonstrated that melittin is digested rapidly by trypsin [46] and is entirely degraded by pepsin after 2 h of incubation [47]. In our study, the resistance of the MDLs to protease was examined, as shown in Figure 5.

As shown in Figure 5A, compared with melittin, the resistance of the MDLs to trypsin increased slowly with the extension of the fatty acid carbon chain length in the range of C2~C10. When the length of the fatty acid carbon chain exceeded ten carbon atoms, the degradation rate of the MDLs was lower than 40% after being incubated in a trypsin environment for 6 h. After incubation with pepsin (Figure 5B), the degradation rate of the MDLs increased slowly with the extension of the fatty acid carbon chain length in the range of C2~C8. When the length of the fatty acid carbon chain exceeded eight carbon atoms, the degradation rate of the MDLs was lower than 20% after being incubated in a pepsin environment for 6 h. The protease resistance assay results suggest that melittin with fatty acids demonstrates significant protease stability.

#### 2.5.2. Serum Stability

**Figure 6:**
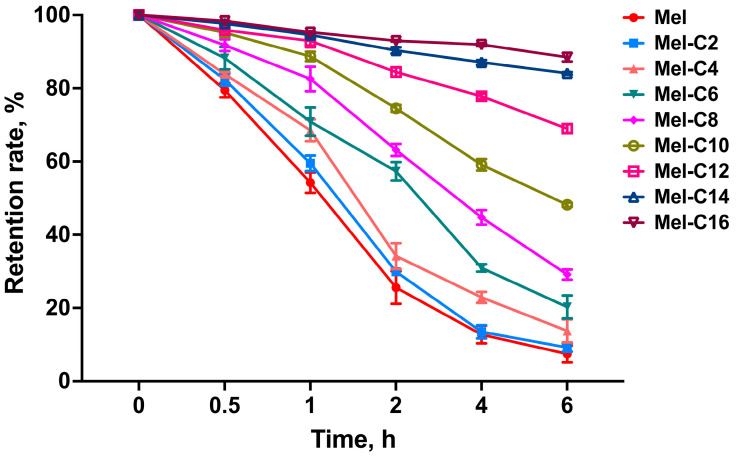
The retention rate of the free MDLs during incubation with piglet serum. Fresh piglet serum was incubated with an equal volume of the MDLs (500 μg/mL) at 37 °C for 6 h. RP-HPLC was used to analyze the retention rate of the free MDLs. Data represent the average ± SEM of three independent experiments.

Earlier studies found that melittin was stable in fetal bovine serum, human serum, and human plasma, indicating that melittin could maintain stability in the presence of serum components [18]. However, binding with serum albumin would weaken the detection of the free MDLs and reduce their antibacterial activity. Here, we evaluated the retention rate of the free MDLs by incubation with piglet serum, and the resulting data are exhibited in Figure 6.

As is shown in Figure 6, with a prolonged incubation time, the retention rate of the MDLs in plasma gradually decreased due to the decrease in free melittin and MDLs after binding with various proteins in the plasma. Moreover, the retention rate of the free MDLs gradually increased with the lengthening of the carbon chain. This is attributed to the fact that fatty acids can prevent MDLs from binding to the proteins in piglet serum, and this was positively correlated with the length of the carbon chain.

#### 2.5.3. Salt Sensitivity

AMPs realize antimicrobial activity by binding to the negative bacterial membrane through electrostatic interaction [48]. Bodily fluids containing high salt concentrations and salt ions under physiological conditions likely affect biological activity. *E. coli* ATCC 25922 and *S. aureus* ATCC 25923 were treated with the MDLs in the presence of different salts using MIC measurements to examine the effects of different physiological saline conditions on their antimicrobial activity.

From the data shown in Table 4, antimicrobial activity was decreased for most of the MDLs under saline conditions, with MIC values increasing by 1~8 times. Compared with K^+^, Mg^2+^, and Fe^3+^, there was no remarkable reduction in the antimicrobial activity of the MDLs in the presence of Na^+^. The free ions can impede the electrostatic interactions between AMPs and anionic bacterial membranes due to a charged shielding effect [49]. It can be inferred that monovalent (K^+^) and divalent (Mg^2+^) ions hinder AMP binding to bacterial membranes, ultimately resulting in decreased antimicrobial activity. Despite the varying degrees of fluctuation in the MICs under physiological saline conditions, the C6 and C8 MDLs were not wholly inactive but retained a relatively desirable active state. This indicates tolerance to the presence of physiological salts.

### 2.6. Antimicrobial Mechanisms of MDLs

#### 2.6.1. Outer Membrane Permeabilization Assay

Assessing the permeabilization in the membranes of natural bacteria is critical to adequately characterizing AMPs. The hydrophobic fluorescent probe N-Phenyl-1-naphthylamine (NPN) can be quenched under aqueous conditions, and its fluorescence intensity is enhanced when released into hydrophobic environments [27]. NPN cannot enter normal bacteria and is taken up once the outer membrane of the bacteria is damaged, leading to an increase in fluorescence intensity. In our study, the ability of the MDLs to permeabilize the outer membrane of *E. coli* ATCC 25922 was tested using NPN uptake assays.

**Figure 7:**
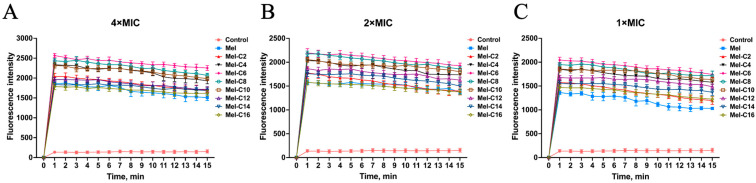
The outer membrane permeabilization of *E. coli* ATCC 25922 induced by different concentrations of MDLs. The permeabilization was assessed using the fluorescence generated by the hydrophobic dye NPN. (**A**) NPN fluorescence detection after treatment with 4 × MIC MDLs; (**B**) NPN fluorescence detection after treatment with 2 × MIC MDLs; (**C**) NPN fluorescence detection after treatment with 1 × MIC MDLs. Data represent the average ± SEM of three independent experiments.

As is shown in Figure 7, after incubation with the MDLs, the fluorescence intensity of NPN rapidly increased within 1 min at concentrations ranging from 1 to 4 × MIC and showed a concentration correlation. This indicates that the MDLs could quickly permeate the outer membrane of *E. coli* ATCC 25922. For all the MDLs, the outer membrane permeabilization was higher than for melittin at the same concentration. These results suggest that the MDLs exhibit a better membrane permeabilization capacity. Previous reports show that AMPs can be electrostatically adsorbed to the membrane surface. Thus, the hydrophobic core of the AMPs can be inserted into the phospholipid layer, which leads to outer membrane rupture through the transmembrane potential [50]. It has been proven that AMPs kill bacteria by disrupting bacterial membranes, which is regarded as an ideal antimicrobial target facing bacterial resistance [51].

#### 2.6.2. Inner Membrane Permeabilization Assay

The enzyme β-galactosidase is located on bacterial inner membranes. It can decompose the molecule 2-Nitrophenyl β-D-galactopyranoside (ONPG), a chromogenic mimic of the natural substrate lactose. It is commonly used to detect inner membrane permeabilization.

**Figure 8:**
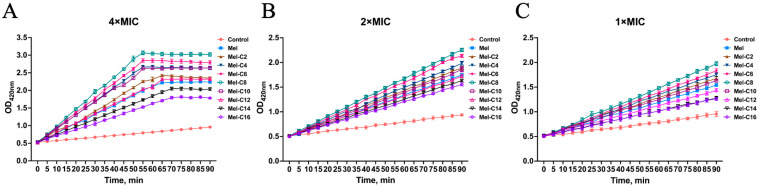
The inner membrane permeabilization of *E. coli* ATCC 25922 induced by different concentrations of the MDLs. Hydrolysis of ONPG due to the release of cytoplasmic β-galactosidase of *E. coli* ATCC25922 treated with varying concentrations of the MDLs was measured spectroscopically at an absorbance of 420 nm and as a function of time. (**A**) OD_420nm_ value detection after treatment with 4 × MIC MDLs; (**B**) OD_420nm_ value detection after treatment with 2 × MIC MDLs; (**C**) OD_420nm_ value detection after treatment with 1 × MIC MDLs. Data represent the average ± SEM of three independent experiments.

Earlier studies have shown that after AMPs permeabilize the outer membranes of Gram-negative bacteria, some of these peptides diffuse through the periplasmic space and then reach the inner membrane. The extent of permeation to the inner membrane intensifies as more AMP molecules arrive [52]. In our study, the MDLs incubated with *E. coli* ATCC 25922 penetrated the inner membrane. Then, the ONPG entered the bacterial cytoplasm and was degraded by β-galactosidase to o-nitrophenol. This was detected at OD420 nm using a microplate reader. As is shown in Figure 8, all of the MDLs increased the inner membrane permeability at 4 × MIC; however, in comparison, the inner membrane permeability of the MDLs was minimally compromised at 1 × MIC. This confirms that while outer membrane damage occurs rapidly and at lower AMP concentrations, the inner membrane requires longer exposures and higher peptide concentrations [53].

#### 2.6.3. LPS/LTA Competitive Inhibition Assay

LPS and LTA are essential to Gram-negative and Gram-positive bacterial membranes, respectively. AMPs with a positive charge target are bound to anionic bacterial membrane surfaces by an electrostatic interaction. The interaction with LPS or LTA is hypothesized to be the first step of the coupling between lipopeptides on bacterial membranes.

**Figure 9:**
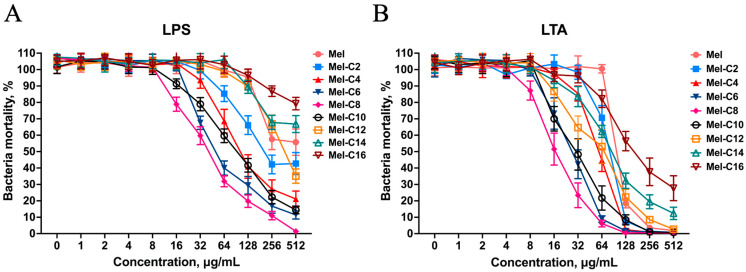
LPS/LTA competitive inhibition of MDLs against *E. coli* ATCC 25922. MDLs were incubated with LPS or LTA at various concentrations (0, 1, 2, 4, 8, 16, 32, 64, 128, 256, and 512 μg/mL) for 30 min at 37 °C, then incubated with *E. coli* ATCC 25922 for 18 h. After incubation, the OD_600 nm_ value of the bacterial culture suspension was measured, and the bacteria mortality was calculated. (**A**) Bacteria mortality of *E. coli* ATCC 25922 after LPS competitive inhibition with MDLs; (**B**) bacteria mortality of *E. coli* ATCC 25922 after LPS competitive inhibition with MDLs. Data represent the average ± SEM of three independent experiments.

LPS or LTA, present in large amounts in the cell envelopes of Gram-positive and Gram-negative bacteria, may interact with MDLs via charge residue, forming a barrier to reduce the concentration of MDLs around the plasma membranes, thereby blunting their antimicrobial effects. As is shown in Figure 9, the MDLs could bind to high concentrations of LPS or LTA and exhibited a competitive binding to LTA rather than LPS. This implies high membrane selectivity towards Gram-negative and Gram-positive bacterial membranes. This explains why the new MDLs exerted better antimicrobial activity against Gram-positive than Gram-negative bacteria. In short, after an initial electrostatic interaction with LPS or LTA, the MDLs were inserted into the bacterial outer and inner lipid membranes. Consequently, they disturbed or destroyed the bacterial membrane, resulting in bacterial death.

## 3. Materials and Methods

### 3.1. Bacterial Strain

The standard strains of *S. aureus* ATCC 43300, *E. faecalis* ATCC 29212, *E. coli* ATCC 25922, and *P. aeruginosa* ATCC 27853 were obtained from the American Type Culture Collection (ATCC). *L. monocytogenes* CVCC 3764 and *B. cereus* CVCC 4101 were obtained from the National Center for Veterinary Culture Collection (CVCC). *S. castellani* CGMCC 1.1869 was obtained from the China General Microbiological Culture Collection Center. The multidrug-resistant strain *Escherichia coli* was obtained from the research base of the Chongqing Academy of Animal Sciences (Chongqing, China). All strains were cultured in nutrient broth overnight at 37 °C to a logarithmic growth phase before each experiment.

### 3.2. Peptide Design, Synthesis, and Analysis

The MDLs were synthesized using solid-phase methods with minor modifications [54]. Fatty acids (acetic acid, butyric acid, caproic acid, caprylic acid, decylic acid, lauric acid, myristic acid, palmitic acid, stearic acid, and eicosanoic acid) (Macklin, Shanghai, China) were linked to the melittin N-terminus using the same method as for amino acid condensation. The final MDLs were purified and analyzed using RP-HPLC (Agilent, Santa Clara, CA, USA) on a PrepHT 300SB-C18 column (21.2 × 250 mm, 7 μm) (Agilent, Santa Clara, CA, USA) and a Kromasil-C18 column (4.6 × 250 mm, 5 μm) (Nouryon, Bohus, Sweden). The MDL identities were confirmed using ESI-MS (Shimadzu, Kyoto, Japan).

### 3.3. Hydrophobicity Assay

Each MDL was dissolved and diluted to 0.1 mg/mL in deionized water (obtained in-house) and then subjected to RP-HPLC on a ChromCore120 C18 column (4.6 × 250 mm, 5 μm) (NanoChrom, Suzhou, China) using 0.1% trifluoroacetic acid (TFA) (Macklin, Shanghai, China) in water as solvent A and 0.1% TFA in acetonitrile (Merck, Darmstadt, Germany) as solvent B. The elution gradient was as follows: 5% B to 90% B over 30 min, 90% B maintained for 2 min, and 90% B to 5% B over 1 min. The flow rate was 1 mL/min and measurements were taken at 220 nm. The retention time was recorded for each MDL.

### 3.4. CD Measurements

The CD spectra of the MDLs were measured at 25 °C on a Chirascan spectrometer (Applied Photophysics, Surrey, UK) using a quartz cell with a 1.0 mm path length, recording from 180 to 260 nm. A scanning speed of 50 nm/min was used and the bandwidth was 1 nm. The optical path and slit width were 0.1 cm and 0.1 nm, respectively. The MDL solutions with a final concentration of 0.5 mg/mL were prepared in deionized water to mimic an aqueous environment, 50% TFE (Macklin, Shanghai, China) was used to mimic the hydrophobic condition of the microbial membrane, and 25 mM SDS (Macklin, Shanghai, China) micelles were used to mimic the negatively charged prokaryotic membrane. The observed ellipticity was converted to molar ellipticity using the following equation:

$$\left[\theta \right]={\left[\theta \right]}_{obs}(MRW/10\times c\times l)$$

where [*θ*] is the molar ellipticity (deg·cm^2^·dmol^−1^), ${\left[\theta \right]}_{obs}$ is the observed ellipticity corrected for the buffer at a given wavelength (mdeg), *MRW* is the mean residual molecular weight (molecular weight/number of amino acids), *c* is the peptide concentration (mg/mL), and *l* is the path length (cm). Assuming lipopeptides were a two-state model, the percentage in the α-helix structure of the MDLs was calculated using the following equation:

$$\alpha \text{-}\mathrm{helical}\;\mathrm{content}=-({\left[\theta \right]}_{222}\;+2000)/30,000\times 100\%$$

### 3.5. MIC Measurements

MIC was determined using the broth microdilution method with minor modification [55]. In brief, bacteria (*S. aureus* ATCC 43300, *E. faecalis* ATCC 29212, *E. coli* ATCC 25922, *P. aeruginosa* ATCC 27853, *L. monocytogenes* CVCC 3764, *B. cereus* CVCC 4101, *S. Castellani* CGMCC 1.1869, and the multidrug-resistant *E. coli* strain) were cultured in nutrient broth (NB) medium (Macklin, Shanghai, China) to the logarithmic phase and diluted to 1 × 10^6^ colony forming units (CFUs)/mL. The MDLs were dissolved to a final concentration of 256~0.25 μg/mL in NB medium using two-fold dilutions. A total of 50 μL of different concentrations of the MDL solutions and an equal volume of bacterial suspension were added to a 96-well plate, which was then incubated at 37 °C for 18 h. The minimum concentrations of the MDLs with no visible growth of bacteria were defined as the corresponding MICs. Media containing bacteria without the MDLs were set as the negative control. Each measurement was performed independently three times.

### 3.6. Hemolytic Activity Assay

The hemolytic activity of the MDLs was evaluated as described previously with minor modification [56]. Swine erythrocytes were collected by centrifugation at 1000 rpm for 5 min, washed three times, and suspended to a final concentration of 8% vol/vol with PBS (Macklin, Shanghai, China). Equal volumes of erythrocyte suspension and the MDLs solutions at various concentrations were mixed in a 96-well plate and incubated for 1 h at 37 °C. After centrifugation at 1000 rpm for 10 min, the supernatant was transferred to a 96-well plate. The supernatant optical density (OD) was measured at 490 nm using a microplate reader (BioTek, Winooski, VT, USA). Untreated erythrocytes and erythrocytes treated with 1% Triton X-100 (Merck, Darmstadt, Germany) were employed as the negative and positive controls, respectively. The hemolysis percentage was calculated using the following equation:

$$\mathrm{Hemolysis}\;\mathrm{rate}\left(\%\right)=\frac{{\mathrm{OD}}_{490(\mathrm{lipopeptide})}-{\mathrm{OD}}_{490(\mathrm{PBS})}}{{\mathrm{OD}}_{490(1\%\;\mathrm{Triton}\;X\text{-}100)}-{\mathrm{OD}}_{490(\mathrm{PBS})}}\times 100\%$$

### 3.7. Stability Assay

#### 3.7.1. Protease Resistance Assay

The resistance of the MDLs to trypsin and pepsin was evaluated based on previous studies with minor modification [57]. After incubation with trypsin (Merck, Darmstadt, Germany) and pepsin (Merck, Darmstadt, Germany), MDL degradation was further determined using RP-HPLC analysis to assess the effect of protease on MDL stability. A total of 500 μg/mL of each MDL was mixed with equal volumes of 0.2 mg/mL of trypsin and pepsin and incubated at 37 °C for 6 h. Aliquots of 50 μL were withdrawn at 0, 0.5, 1, 2, 4, and 6 h and incubated at 60 °C for 5 min to terminate the reaction. Then, samples were centrifugated at 13,000 rpm for 15 min, and RP-HPLC was used to detect the enzymatic digestion of the MDLs. The samples were gradient eluted using 5~95% acetonitrile/water in 0.1% TFA at a flow rate of 1 mL/min within 30 min, and the ultraviolet absorbance was set as 220 nm.

#### 3.7.2. Serum Stability

The serum stability of the MDLs was analyzed using RP-HPLC and a protease resistance assay. Fresh piglet serum was harvested and incubated with equal volumes of the MDLs (500 μg/mL) at 37 °C for 6 h. At 0, 0.5, 1, 2, 4, and 6 h, aliquots of the samples were withdrawn, and the degradation reaction was terminated by acetonitrile. Then, the samples were centrifugated at 13,000 rpm for 15 min, and RP-HPLC was used to analyze the supernatants.

#### 3.7.3. Salt Sensitivity

MIC determinations of *S. aureus* ATCC 43300 and *E. coli* ATCC 25922 were performed at physiological salt concentrations (150 mM NaCl, 4.5 mM KCl, 1 mM MgCl_2_, and 4 mM FeCl_3_) (Macklin, Shanghai, China) as described above to evaluate the effect of salt on the bacteriostatic activity of the MDLs. This was carried out in triplicate and tested at least twice.

### 3.8. Antimicrobial Mechanism

#### 3.8.1. Outer Membrane Permeabilization Assay

The outer membrane permeabilization of the MDLs was measured using a fluorescent dye NPN (Merck, Darmstadt, Germany) assay. Briefly, *E. coli* ATCC 25922 was cultured to the logarithmic phase in NB medium and diluted to OD600 nm = 0.5. After centrifugation at 8500 rpm for 5 min, the bacterial precipitation was washed three times with PBS and suspended with half the volume in the same buffer. The MDLs were dissolved in PBS to final concentrations of 4, 2, and 1 × MIC and were added to a black 96-well plate. The bacterial suspension was added after mixing with NPN dye (40 mM). Changes in fluorescence were recorded using a microplate reader within 15 min (excitation = 350 nm, emission = 420 nm). PBS without MDLs was used as a negative control, and PBS without MDLs or bacteria was used as a blank control. The measured values were converted to % NPN uptake using the following equation:

$$\mathrm{NPN}\;\mathrm{uptake},\;\%=\frac{F_{t}-F_{0}}{F_{m}-F_{0}}\times 100$$

where *F_t_* is the fluorescence at different MDL concentrations; *F*_0_ is the initial fluorescence of NPN in the absence of MDL, as a negative control; and *F_m_* is the fluorescence when polymyxin B was added, as a positive control.

#### 3.8.2. Inner Membrane Permeabilization Assay

The inner membrane permeabilization of bacteria treated with the MDLs was determined by measuring the release activity of bacterial cytoplasm galactosidase utilizing ONPG as a substrate. *E. coli* ATCC 25922 was cultured in NB medium to the logarithmic phase and diluted to OD 420 nm = 0.6. Bacteria were harvested by centrifugation at 1500 rpm for 10 min, washed three times with PBS, and suspended with half the volume in the same buffer. ONPG (30 mM) (Merck, Darmstadt, Germany) was added to a 96-well plate with different concentrations of MDLs, and the bacterial suspension was added to give final MDL concentrations of 4, 2, and 1 × MIC, respectively. PBS without MDL was used as the negative control, and 1% TritonX-100 was used as the positive control. The OD values at 420 nm, detected using a microplate reader, indicated inner membrane permeabilization.

#### 3.8.3. LPS/LTA Competitive Inhibition Assay

Newly synthesized MDLs were incubated with LPS (Merck, Darmstadt, Germany) or LTA (Merck, Darmstadt, Germany) at various concentrations for 30 min at 37 °C and then incubated with *E. coli* ATCC 25922 for 18 h. The final concentrations of the MDLs were modulated to 1 × MIC and the LPS/LTA final concentrations varied from 1 to 512 mg/mL. After incubation, the OD values of the bacterial culture suspensions were measured using a microplate reader at 600 nm. Three independent experiments were performed in triplicate.

## 4. Conclusions

In this study, we synthesized a series of new MDLs using an N-terminal fatty acid conjugation approach. Most of the MDLs showed perfect antimicrobial potential against Gram-positive bacteria, including the multidrug-resistant *E. coli* strain. Among the MDLs, C8-Mel presented the highest antimicrobial selectivity with low cytotoxicity and maintained the best antimicrobial activity in the presence of physiological salts and serum. Most notably, with their unique membrane mechanisms, all MDLs could bind to LPS or LTA; they can kill bacteria by increasing the outer and inner membrane permeability and damaging membrane integrity, leading to intracellular content leakage. Although the C8-Mel shows satisfactory antibacterial activity and enzymatic stability, its hemolysis needs further improvement. In addition, the mammalian cytotoxicity, antiviral, and anticancer activities of the MDLs need to be deeply investigated in our future research. Taken together, fatty acylation of melittin could be an effective method for enabling potential therapeutic use. The newly synthesized C8-Mel is a novel antimicrobial candidate for fighting the increasing antibiotic resistance of Gram-positive bacteria.

## Figures and Tables

**Table 1:** The main physicochemical properties of MDLs.

Peptide	Sequence	Theoretical MW	Measured MW ^a^	Net Charge	RT, Min ^b^
Mel	GIGAVLKVLTTGLPALISWIKRKRQQ	2846.46	2846.15	+6	8.076 ± 0.014
C2-Mel	C2-GIGAVLKVLTTGLPALISWIKRKRQQ	2889.50	2889.45	+6	12.247 ± 0.021
C4-Mel	C4-GIGAVLKVLTTGLPALISWIKRKRQQ	2917.56	2917.65	+6	13.704 ± 0.005
C6-Mel	C6-GIGAVLKVLTTGLPALISWIKRKRQQ	2945.61	2945.70	+6	15.567 ± 0.006
C8-Mel	C8-GIGAVLKVLTTGLPALISWIKRKRQQ	2973.66	2973.80	+6	17.845 ± 0.013
C10-Mel	C10-GIGAVLKVLTTGLPALISWIKRKRQQ	3001.71	3001.60	+6	20.541 ± 0.029
C12-Mel	C12-GIGAVLKVLTTGLPALISWIKRKRQQ	3029.77	3029.70	+6	23.344 ± 0.004
C14-Mel	C14-GIGAVLKVLTTGLPALISWIKRKRQQ	3057.82	3057.6	+6	25.621 ± 0.002
C16-Mel	C16-GIGAVLKVLTTGLPALISWIKRKRQQ	3085.87	3085.7	+6	26.892 ± 0.005

^a^ Molecular weight (MW) was measured using electrospray ionization-mass spectrometry (ESI-MS). ^b^ Analytical RP-HPLC was used to determine retention time on a C18 column.

**Table 2:** Percentage α-helical and β-fold contents of melittin and the MDLs in different environments.

Peptide	PBS Buffer		50% TFE		25 mM SDS	
	α-Helix, %	β-Fold, %	α-Helix, %	β-Fold, %	α-Helix, %	β-Fold, %
Mel	6.8	38.5	34.5	5.5	21.5	17.9
C2-MEL	6	32.8	26.5	17.8	20.9	19.9
C4-MEL	6.1	33.7	20.7	25.4	19.3	23.8
C6-MEL	6.5	39.3	22.4	27	18.4	24.7
C8-MEL	7	40	18.8	25.5	18.2	25.4
C10-MEL	7.5	41.1	18.4	22.1	17.1	28.8
C12-MEL	11.5	31.3	19	21.5	15.9	29.5
C14-MEL	12	31	16	25.2	15.5	31.7
C16-MEL	12.5	30.4	14.3	26.8	15.2	33

**Table 3:** Antimicrobial activity of the MDLs against standard and multidrug-resistant strains.

Peptide	MIC, μg/mL						
	Gram Positive				Gram Negative		
	*Staphylococcus aureus* (*S. aureus*) ATCC 43300	*Listeria monocytogenes* (*L. monocytogenes*) CVCC 3764	*Enterococcus faecalis* (*E. faecalis*) ATCC 29212	*Bacillus cereus* (*B. cereus*) CVCC 4101	*Escherichia coli* (*E. coli*) ATCC 25922	*Shigella castellani* (*S. castellani*) CGMCC 1.1869	Multidrug-Resistant *Escherichia coli* (*E. coli*) Strain
Mel	8	32	16	16	32	32	64
C2-Mel	4	32	4	8	16	8	64
C4-Mel	2	8	2	8	8	8	32
C6-Mel	1	4	2	4	4	8	8
C8-Mel	0.5	4	1	2	2	4	4
C10-Mel	2	16	8	8	8	32	8
C12-Mel	8	32	16	64	32	64	32
C14-Mel	32	128	32	>256	128	128	128
C16-Mel	64	>256	64	>256	>256	128	>256

ATCC, American Type Culture Collection; CVCC, China Veterinary Culture Collection Center; CGMCC, China General Microbiological Culture Collection Center.

**Table 4:** MICs of the MDLs against *E. coli* ATCC 25922 and *S. aureus* ATCC 43300 in the presence of physiological salts.

Bacterial Strain	Peptide	Control	Salt			
			NaCl	KCl	MgCl_2_	FeCl_3_
*E. coli* ATCC 25922	Mel	32	32	32	64	64
	Mel-C2	16	16	16	32	32
	Mel-C4	8	8	16	32	32
	Mel-C6	4	4	8	16	16
	Mel-C8	2	2	8	8	8
	Mel-C10	8	8	16	32	32
	Mel-C12	16	32	32	128	128
	Mel-C14	32	128	64	256	256
	Mel-C16	128	>256	256	>256	>256
*S. aureus* ATCC 43300	Mel	8	8	8	32	32
	Mel-C2	4	4	4	16	16
	Mel-C4	2	2	4	16	16
	Mel-C6	1	1	2	8	8
	Mel-C8	0.5	0.5	1	8	8
	Mel-C10	2	2	8	32	32
	Mel-C12	8	16	32	64	64
	Mel-C14	32	64	64	256	256
	Mel-C16	64	128	256	>256	>256

## Data Availability

Data are contained within the article.
